# Retraction: Clinical and Functional Outcomes of Telescoping Intramedullary Nails in Pediatric Osteogenesis Imperfecta: A Multicenter Prospective Study With a One-Year Follow-Up

**DOI:** 10.7759/cureus.r250

**Published:** 2026-10-08

**Authors:** Muhammad Tayyab, Muhammad Saqib, Muhammad Tanveer, Zawar Ahmad, Asif Afridi, Muhammad Arsalan Azmat Swati

**Affiliations:** 1 Trauma and Orthopaedics, Bradford Teaching Hospitals, Bradford, GBR; 2 Orthopaedic Surgery, Gajju Khan Medical College Swabi, Swabi, PAK; 3 Trauma and Orthopaedics, University Hospitals of North Midlands, Stoke-on-Trent, GBR; 4 Department of Trauma and Orthopaedics, Kettering General Hospital NHS Trust, Kettering, GBR; 5 Department of Trauma and Orthopaedics, Hayatabad Medical Complex Peshawar, Peshawar, PAK; 6 Department of Trauma and Orthopaedics, University Hospital Birmingham, Birmingham, GBR; 7 Department of Orthopaedics, Medical Teaching Institution (MTI) Mardan Medical Complex, Mardan, PAK

This article has been retracted by the Editor-in-Chief due to concerns regarding the reliability of the reported study data. After publication, the authors identified an inconsistency in the data and requested that the article be retracted. Retraction was deemed appropriate upon confirmation of the data inconsistencies. All authors agree with the decision to retract.

